# Lanthanum (La) improves growth, yield formation and 2-acetyl-1-pyrroline biosynthesis in aromatic rice (*Oryza sativa* L.)

**DOI:** 10.1186/s12870-021-03006-5

**Published:** 2021-05-25

**Authors:** Haowen Luo, Yulin Chen, Longxin He, Xiangru Tang

**Affiliations:** 1 State Key Laboratory for Conservation and Utilization of Subtropical Agro-Bioresources, College of Agriculture, South China Agricultural University, Guangzhou, 510642 China; 2 Scientific Observing and Experimental Station of Crop Cultivation in South China, Ministry of Agriculture and Rural Affairs, Guangzhou, 510642 China; 3 Guangzhou Key Laboratory for Science and Technology of Aromatic Rice, Guangzhou, 510642 China; 4 The College of Natural Resources and Environment, College of Agriculture, South China Agricultural University, Guangzhou, 510642 China

**Keywords:** 2-acetyl-1-pyrroline, Aromatic rice, Lanthanum, Photosynthesis, Proline, Yield formation

## Abstract

**Background:**

Lanthanum (La) is a rare earth element that can influence plant growth and development. However, the effect of La on growth, yield formation and 2-acetyl-1-pyrroline (2-AP, a key compound responsible for the aroma of rice) biosynthesis in aromatic rice (*Oryza sativa* L. subsp. *japonica* Kato) has not been reported. Therefore, the present study investigated the effects of La on growth, photosynthesis, yield formation and 2-AP biosynthesis in aromatic rice through three experiments.

**Results:**

Two pot experiments and a two-year field trial were conducted with different rates of La application (20–120 LaCl_3_ mg kg^−1^ and 12 kg ha^−1^ LaCl_3_), and treatments without La application were used as controls. The results showed that the application of LaCl_3_ at 80 and 100 mg kg^−1^ and at 12 kg ha^−1^ greatly increased the 2-AP content (by 6.45–43.03%) in aromatic rice seedlings and mature grains compared with the control. The La treatments also increased the chlorophyll content, net photosynthetic rate and total aboveground biomass of rice seedlings. Higher antioxidant enzyme (superoxide, peroxidase, and catalase) activity was detected in the La treatments than in the control. The La treatments also increased the grain yield, grain number per panicle and seed-setting rate of aromatic rice relative to the control. Moreover, the grain proline and γ-aminobutyric acid contents and the activity of betaine aldehyde dehydrogenase significantly decreased under the La treatment. The application of La to soil enhanced the activity of proline dehydrogenase by 20.62–56.95%.

**Conclusions:**

La improved the growth, yield formation and 2-AP content of aromatic rice and enhanced 2-AP biosynthesis by increasing the conversion of proline to 2-AP and decreasing the conversion of GABald to GABA.

## Background

Aromatic rice (*Oryza sativa* L.) is well known worldwide for its characteristic aroma and is also highly desired by consumers, attracts premium prices in many international markets [1–4]. The aromatic compounds of aromatic rice are very complicated, and more than 200 volatile substances were detected in aromatic rice in previous studies [5]. In recent years, it has been clearly established that 2-acetyl-1-pyrroline (2-AP) is the key flavor compound in aromatic rice that imparts its characteristic aroma [6, 7].

The biosynthesis of 2-AP in aromatic rice is a relatively clear process. Previous studies discovered that 2-AP formation is closely related to proline and γ-aminobutyric acid (GABA). In 2002, Yoshihashi et al. [8] revealed that proline is the nitrogen source for 2-AP through an isotope tracing test. Mo et al. [4] showed that 2-AP content is positively correlated with both the proline and GABA in aromatic rice. Chen et al. [9] demonstrated that 2-AP biosynthesis in aromatic rice is inhibited by the expression of the *BADH2* gene; this gene encodes betaine aldehyde dehydrogenase (BADH) which catalyzes the conversion of γ-aminobutyl aldehyde (GABald) into GABA instead of 2-AP. Furthermore, the study of Mo et al. [10] indicated that the 2-AP in aromatic rice was transformed mainly from proline catalyzed by proline dehydrogenase (PDH).

Lanthanum (La) is one of the rare earth elements, a group that includes 17 elements with similar physical and chemical properties [11]. An earlier study showed that exogenous La significantly ameliorated copper toxicity in rice by reducing oxidative stress and increasing the chlorophyll content [12]. Liang et al. [13] discovered that La application remarkably enhanced plasma membrane H + -ATPase activity in rice under acid rain stress. Liu et al. [14] indicated that exogenous La^3+^ induced regulation by the antioxidant system and affected the concentrations of hydrogen peroxide, superoxide anion, and malondialdehyde in rice roots. Wang et al. [15] also demonstrated that the combination of LaCl_3_ and acid rain substantially increased the net photosynthetic rate, stomatal conductance, Hill reaction activity and carboxylation efficiency of rice plants. Hence, it can be concluded that La has multiple effects on rice growth and development.

In 2016, Mo et al. [10] showed that supplementation with La in basic culture medium enhanced the activity of PDH and increased the 2-AP content in detached aromatic rice panicles in *Vitro*. La might have the ability to enhance 2-AP biosynthesis and the potential to be used in aromatic rice production to cultivate highly aromatic rice. However, no additional studies of the effects of La on aromatic rice growth performance have been published. Furthermore, the mechanism underlying the regulation of 2-AP formation under exogenous La application remains unexplored.

Therefore, the present study was conducted with the hypothesis that La could increase grain yield and 2-AP content in aromatic rice and with the objective of studying the mechanism underlying the effects of La on 2-AP biosynthesis in aromatic rice.

## Results

### Effects of La application on the performance of aromatic rice seedlings

**Fig. 1:**
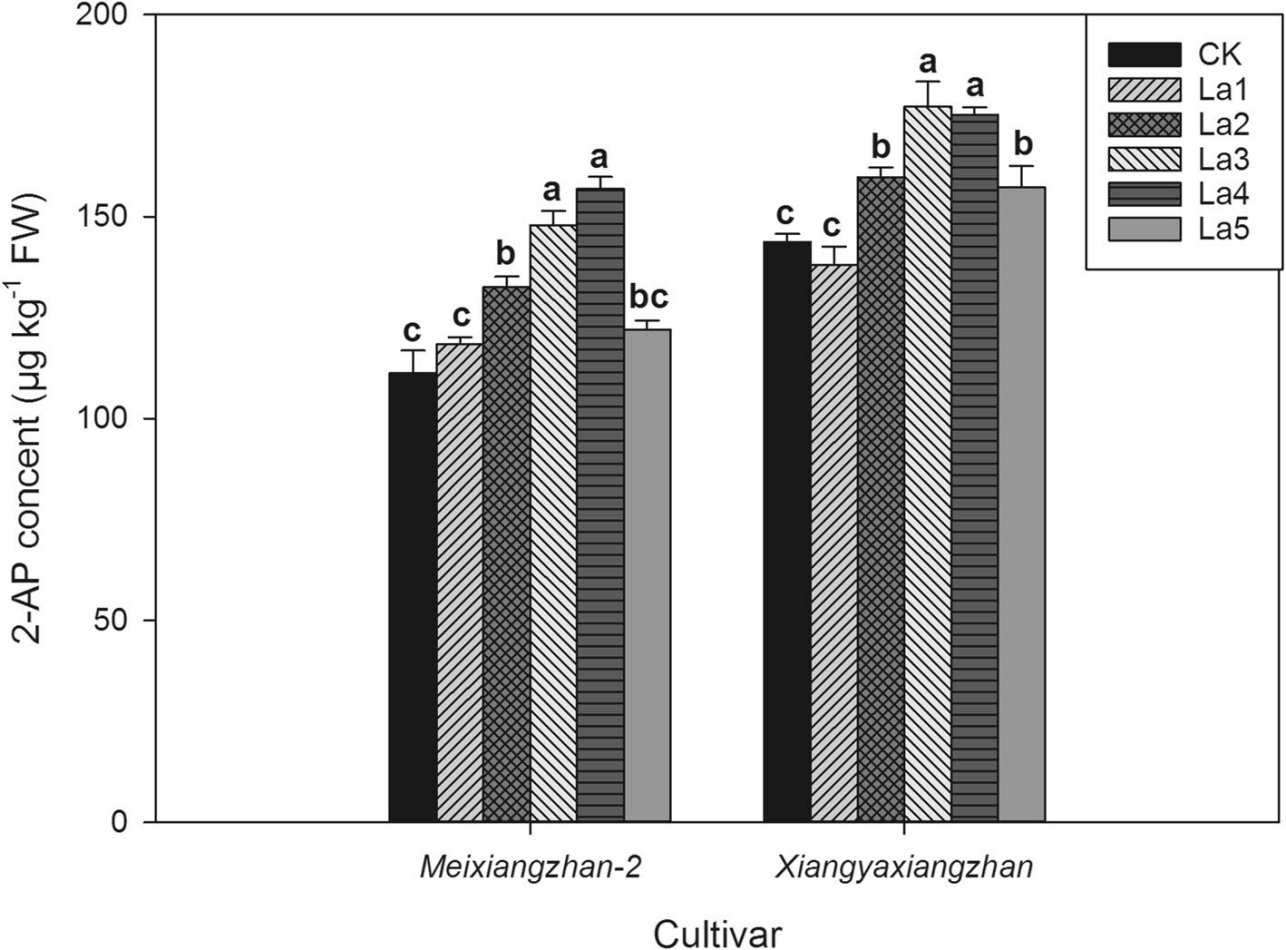
Effects of different La treatments on the 2-AP content of aromatic rice seedlings. Values (means ± SEs) of each treatment were obtained from three independent replications (n = 3). Different letters indicate significant differences among the treatments (*P* < 0.05, least significant difference test)

The application of La to soil significantly influenced the 2-AP content of the aromatic rice seedlings (Fig. 1). Compared with CK, the La treatments (La2, La3 and La4 treatments) notably improved the 2-AP content, by 11.10–41.01% and 11.10–23.38% for *Meixiangzhan-2* and *Xiangyaxiangzhan*, respectively and the highest contents were recorded in the La3 and La4 treatments. In addition, we observed that the La5 treatments suddenly decreased 2-AP content for *Meixiangzhan-2* and *Xiangyaxiangzhan* compared with that in the La3 and La4 treatments. This may have occurred because the content of La in soil in the La5 treatment exceeded the suitable range for fragrant rice and caused metal toxicity. Regarding seedling quality was concerned, the aromatic rice seedlings grown in La containing soils exhibited significant improvements in fresh weight, dry weight and stem diameter compared to those grown in soil without La (Table 1). In comparison with CK, La3 and La4 treatments exhibited significantly higher fresh weight (by 6.00–13.68%), dry weight (by 5.98–19.45%) and stem diameter by (5.43–9.74%).

**Table 1:** Effect of different La treatments on the quality of aromatic rice seedlings

Cultivar	Treatment	Fresh weight (mg)	Dry weight (mg)	Plant Height (cm)	Stem diameter (mm)
*Meixiangzhan-2*					
	CK	168.37 ± 5.61b	27.63 ± 5.94b	20.68 ± 0.94b	2.20 ± 0.34bc
	La1	164.23 ± 1.74b	27.13 ± 6.33b	21.52 ± 0.13b	2.14 ± 1.33bc
	La2	168.60 ± 2.64b	27.14 ± 4.53b	21.45 ± 0.87b	2.11 ± 0.68c
	La3	178.47 ± 3.52ab	33.00 ± 2.40a	26.29 ± 0.46a	2.39 ± 1.17a
	La4	189.87 ± 3.08a	31.37 ± 4.43a	25.15 ± 0.78a	2.32 ± 0.98a
	La5	169.00 ± 2.22b	27.55 ± 4.72b	21.48 ± 1.32b	2.22 ± 0.58b
*Xiangyaxiangzhan*					
	CK	137.20 ± 1.95b	25.02 ± 2.21 cd	24.54 ± 0.98ab	1.85 ± 0.80c
	La1	135.80 ± 4.43b	24.29 ± 6.81d	23.55 ± 0.11b	1.97 ± 1.33ab
	La2	136.33 ± 2.48b	26.67 ± 1.63b	24.51 ± 0.91ab	1.90 ± 1.12bc
	La3	154.73 ± 6.12a	29.67 ± 1.69a	25.35 ± 0.16ab	2.03 ± 0.04a
	La4	155.97 ± 1.87a	26.52 ± 4.44bc	26.98 ± 0.30a	2.03 ± 1.02a
	La5	137.10 ± 5.25b	25.16 ± 6.43bcd	25.41 ± 0.11ab	1.95 ± 0.34abc

Values (means ± SEs) of each treatment were obtained from three independent replications (n = 3). Different letters indicate significant differences among the treatments (*P* < 0.05, least significant difference test)

### Effects of La application on yield and yield related traits of aromatic rice

The yield and yield-related traits of aromatic rice were affected by La application in both the second and third experiments (Table 2 & Table 3). For *Meixianghan-2* in the second experiment, compared with CK, the La80 treatment significantly increased the grain yield (by 12.22%) while the La80 and La100 treatments significantly increased the grain number per panicle, seed-setting rates and total yield. For *Xiangyaxiangzhan* in the second experiment, compared with CK, the La80 and La100 treatments significantly increased the grain yield and total yield by 9.33–10.34% and 15.16–17.19%, respectively. Compared with CK, the La100 and La80 treatments also significantly increased the grain number per panicle and seed-setting rate, respectively. In the field (the third Experiment), in comparison with CK, the La treatment significantly increased grain yield by 3.84–10.73% in 2018 and 2019 for both *Meixiangzhan-2* and *Xiangyaxiangzhan*. Compared with CK, the La treatment also significantly increased the grain number per panicle, by 9.28–19.48%. Moreover, slightly higher seed-setting rates were recorded in the La treatment than in CK in both years and for both cultivars. However, there was no significant difference between the CK and La treatments in the either effective panicle number or the 1000-grain weight.

**Table 2:** Effects of La application on grain yield, effective panicle number, grain number per panicle, seed-setting rate, 1000-grain weight and total yield of aromatic rice in the second experiment

Cultivar	Treatment	Grain yield (g pot^−1^)	Effective panicle number per pot	Grain number per panicle	Seed-setting rate (%)	1000-grain weight (g)	Total yield (g pot^−1^)
*Meixiangzhan-2*							
	CK	53.46 ± 2.18b	28.67 ± 1.03a	133.12 ± 0.39c	71.31 ± 0.21b	19.92 ± 0.03a	19.68 ± 0.04b
	La40	56.32 ± 1.01ab	27.00 ± 2.84a	130.23 ± 0.50bc	75.46 ± 0.56a	20.08 ± 0.02a	18.63 ± 0.02b
	La80	59.99 ± 1.30a	27.67 ± 0.81a	138.81 ± 1.13ab	75.33 ± 0.44a	19.86 ± 0.03a	22.63 ± 0.06a
	La100	58.08 ± 0.65ab	29.67 ± 1.93a	141.83 ± 0.85a	72.24 ± 0.22b	20.18 ± 0.03a	22.31 ± 0.04a
*Xiangyaxiangzhan*							
	CK	53.94 ± 1.74bc	28.33 ± 1.69a	129.83 ± 0.36b	71.40 ± 0.39b	20.47 ± 0.05a	21.35 ± 0.07b
	La40	53.17 ± 0.38c	26.67 ± 2.44a	137.03 ± 0.44ab	72.67 ± 0.36ab	20.10 ± 0.03a	22.09 ± 0.02b
	La80	59.52 ± 1.71a	28.67 ± 2.16a	135.95 ± 0.42ab	75.04 ± 0.28a	20.08 ± 0.04a	24.89 ± 0.02a
	La100	58.97 ± 1.35ab	28.33 ± 2.59a	140.75 ± 0.97a	73.49 ± 0.73ab	20.07 ± 0.01a	25.36 ± 0.04a

Values (means ± SEs) of each treatment were obtained from three independent replications (n = 3). Different letters indicate significant differences among the treatments (*P* < 0.05, least significant difference test)

**Table 3:** Effects of La application on grain yield, effective panicle number, grain number per panicle, seed-setting rate and 1000-grain weight of aromatic rice in the third experiment

Year	Cultivar	Treatment	Grain yield (g pot^−1^)	Effective panicle number per pot	Grain number per panicle	Seed-setting rate (%)	1000-grain weight (g)
2018	*Meixiangzhan-2*						
		CK	5.14 ± 0.09b	260.94 ± 21.33a	132.91 ± 7.69b	77.82 ± 3.68a	20.61 ± 0.05a
		La	5.54 ± 0.10a	295.59 ± 26.74a	158.80 ± 5.16a	76.89 ± 4.19a	20.69 ± 0.04a
	*Xiangyaxiangzhan*						
		CK	4.95 ± 0.18b	257.04 ± 1.00a	141.05 ± 4.92b	77.96 ± 1.25a	19.96 ± 0.40a
		La	5.14 ± 0.17a	269.80 ± 7.77a	154.14 ± 7.45a	77.02 ± 2.18a	20.54 ± 0.15a
2019	*Meixiangzhan-2*						
		CK	5.21 ± 0.08b	260.24 ± 5.13a	143.40 ± 2.38b	77.12 ± 0.89a	20.14 ± 0.11a
		La	5.69 ± 0.07a	287.22 ± 13.00a	158.88 ± 3.08a	78.31 ± 1.92a	20.39 ± 0.46a
	*Xiangyaxiangzhan*						
		CK	4.69 ± 0.18b	267.97 ± 22.61a	138.61 ± 8.06a	78.46 ± 1.47a	20.00 ± 0.44a
		La	5.19 ± 0.06a	288.07 ± 11.59a	153.06 ± 7.72a	77.24 ± 1.07a	19.69 ± 0.06a

Values (means ± SEs) of each treatment were obtained from three independent replications (n = 3). Different letters indicate significant differences among the treatments (*P* < 0.05, least significant difference test)

### Effects of La application on the chlorophyll content of aromatic rice

**Fig. 2:**
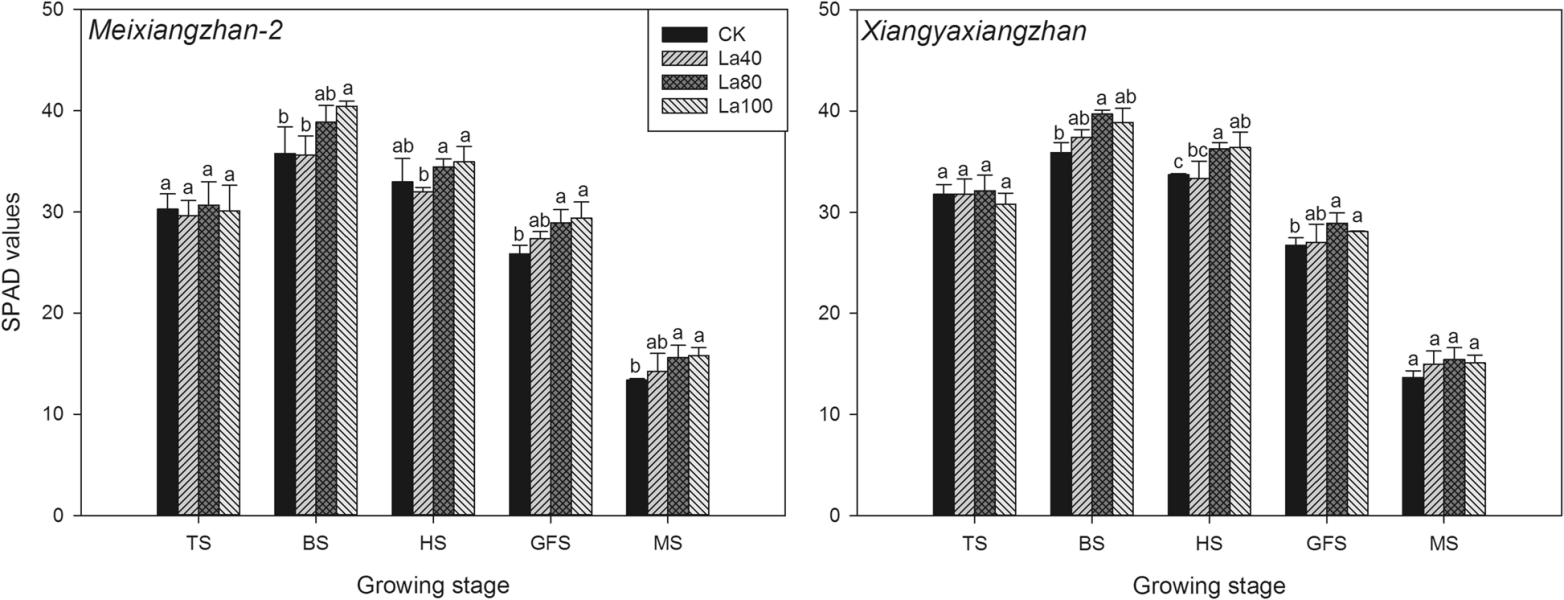
Effects of different La treatments on the SPAD values of aromatic rice in the second experiment. TS: tillering stage; BS: booting stage: HS: heading stage: GFS: grain-filling stage; MS: Maturity stage. Values (means ± SEs) of each treatment were obtained from three independent replications (n = 3). Different letters indicate significant differences among the treatments (P < 0.05, least significant difference test)

**Fig. 3:**
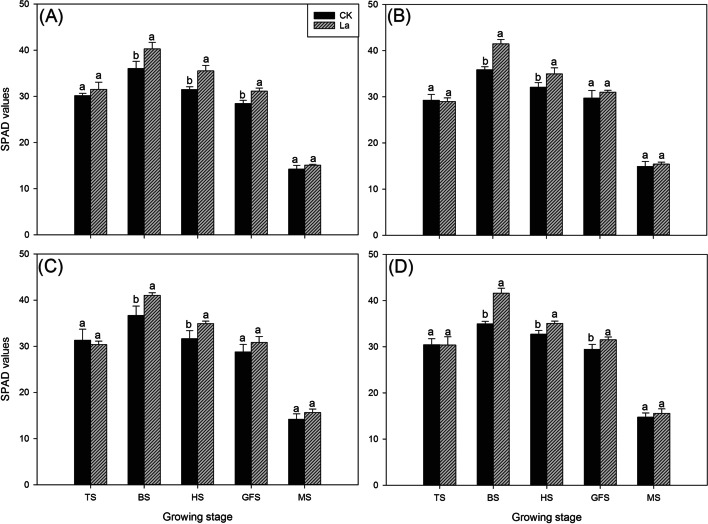
Effects of different La treatments on the SPAD values of aromatic rice in the third experiment. **a ***Meixiangzhan* in 2018; **b ***Xiangyaxiangzhan* in 2018; **c ***Meixiangzhan* in 2019; **d ***Xiangyaxiangzhan* in 2019. TS: tillering stage; BS: booting stage: HS: heading stage: GFS: grain-filling stage; MS: Maturity stage. Values (means ± SEs) of each treatment were obtained from three independent replications (n = 3). Different letters indicate significant differences among the treatments (*P* < 0.05, least significant difference test)

La application substantially increased the chlorophyll content of aromatic rice (Fig. 2 & Fig. 3). In second experiment, there were no significant differences in SPAD values among all the treatments including CK at the tillering stage. At the booting stage, heading stage and grain-filling stage, the La80 and La100 treatments increased the SPAD value by 7.45–13.57% compared with CK, and at the maturity stage, slightly higher SPAD values were recorded in the La80 and La100 treatment than CK. In the third experiment, 4.32–18.95% higher SPAD values were recorded in the La treatment than in the CK treatment at the booting stage, heading stage and grain-filling stage. Compared with CK, the La treatment also slightly increased the SPAD value at the maturity stage.

### Effects of La application on the net photosynthetic rate of aromatic rice

**Fig. 4:**
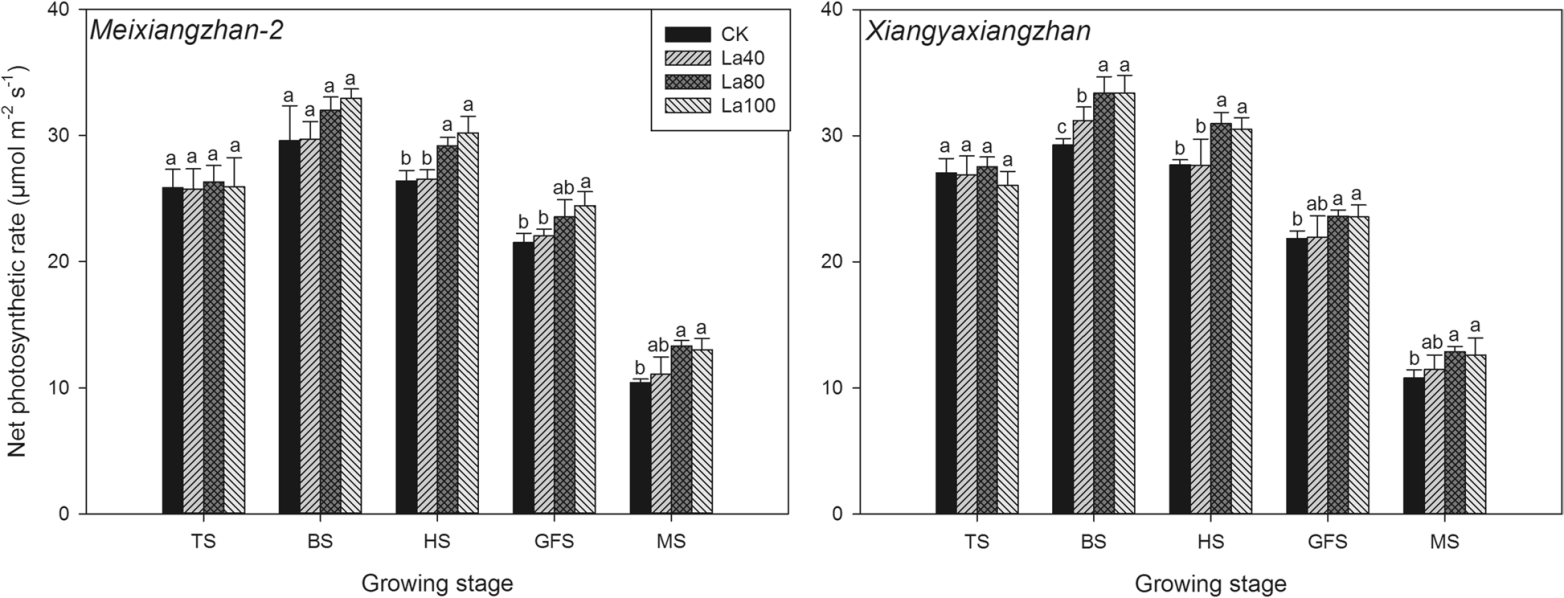
Effects of different La treatments on the net photosynthetic rate of aromatic rice in the second experiment. TS: tillering stage; BS: booting stage: HS: heading stage: GFS: grain-filling stage; MS: Maturity stage. Values (means ± SEs) of each treatment were obtained from three independent replications (n = 3). Different letters indicate significant differences among the treatments (*P* < 0.05, least significant difference test)

**Fig. 5:**
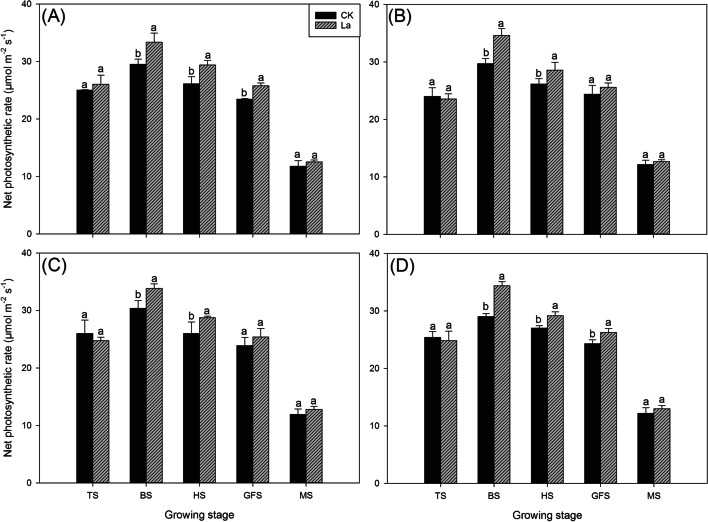
Effects of different La treatments on the net photosynthetic rate of aromatic rice in the third experiment. **a** *Meixiangzhan* in 2018; **b** *Xiangyaxiangzhan* in 2018; **c** *Meixiangzhan* in 2019; **d** *Xiangyaxiangzhan* in 2019. TS: tillering stage; BS: booting stage: HS: heading stage: GFS: grain-filling stage; MS: maturity stage. Values (means ± SEs) of each treatment were obtained from three independent replications (n = 3). Different letters indicate significant differences among the treatments (*P* < 0.05, least significant difference test)

La application substantially improved the net photosynthetic rate of aromatic rice (Fig. 4 & Fig. 5). In the second experiment, there were no significant differences in net photosynthetic rate among all the treatments including CK at the tillering stage. At the booting stage, heading stage and grain-filling stage, the La80 and La100 treatments improved the net photosynthetic rate by 7.45–14.08% compared with that in CK, and at the maturity stage, slightly higher net photosynthetic rates were recorded in the La80 and La100 treatments than in CK. In the third experiment, 4.98–18.41% higher net photosynthetic rates were recorded in the La treatment than CK at the booting stage, heading stage and grain-filling stage. Compared with CK, the La treatment also slightly improved net photosynthetic rate at the maturity stage.

### Effects of La application on dry matter accumulation in aromatic rice

**Fig. 6:**
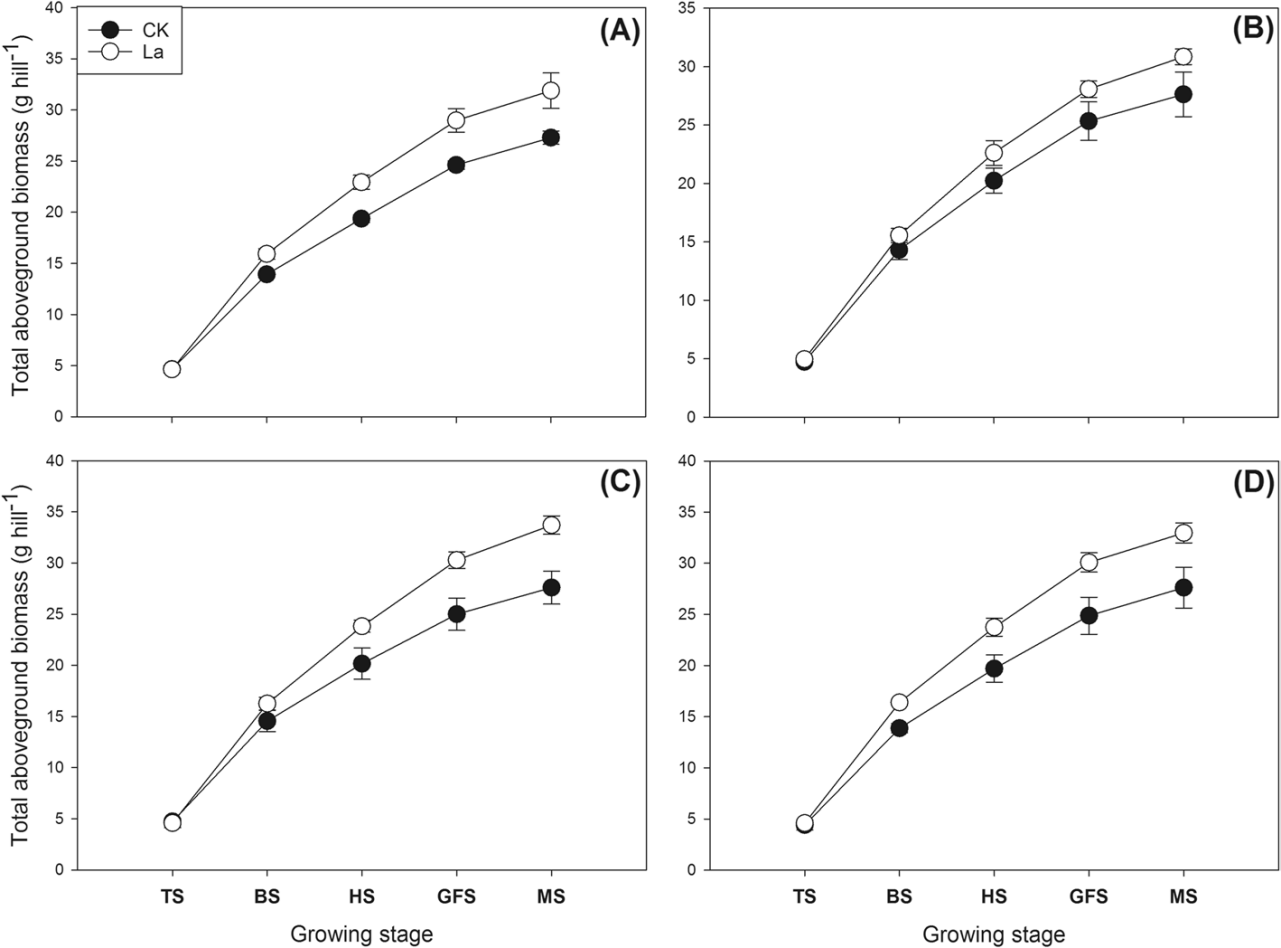
Effects of La application on the total aboveground biomass of aromatic rice in the third experiment. **a ***Meixiangzhan* in 2018; **b ***Xiangyaxiangzhan* in 2018; **c ***Meixiangzhan* in 2019; **d ***Xiangyaxiangzhan* in 2019. TS: tillering stage; BS: booting stage: HS: heading stage: GFS: grain-filling stage; MS: maturity stage. Values (means ± SEs) of each treatment were obtained from three independent replications (n = 3)

La application remarkably enhanced the dry matter accumulation in aromatic rice in the field experiment (Fig. 6). For *Meixangzhan-2* in 2018, 14.33, 18.41, 17.69 and 16.83% higher total aboveground biomasses were recorded in the La treatment than in CK at the booting stage, heading stage, grain-filling stage and maturity stage, respectively. For *Xiangyaxiangzhan* in 2018, 8.74, 11.76, 10.79 and 11.66% higher total aboveground biomasses were recorded in the La treatment than in CK at the booting stage, heading stage, grain-filling stage and maturity stage, respectively. For *Meixiangzhan-2* in 2019, compared with CK, the La treatment significantly increased the total aboveground biomass by 11.69, 18.15, 21.15 and 22.14% in the four growth stages, respectively; for *Xiangyaxiangzhan* in 2019, compared with CK, the La treatment significantly increased the total aboveground biomass by 18.12, 20.53, 20.96 and 19.40% in the four growth stages, respectively.

### Effects of La application on the activities of SOD, POD, and CAT and content of MDA in aromatic rice

**Fig. 7:**
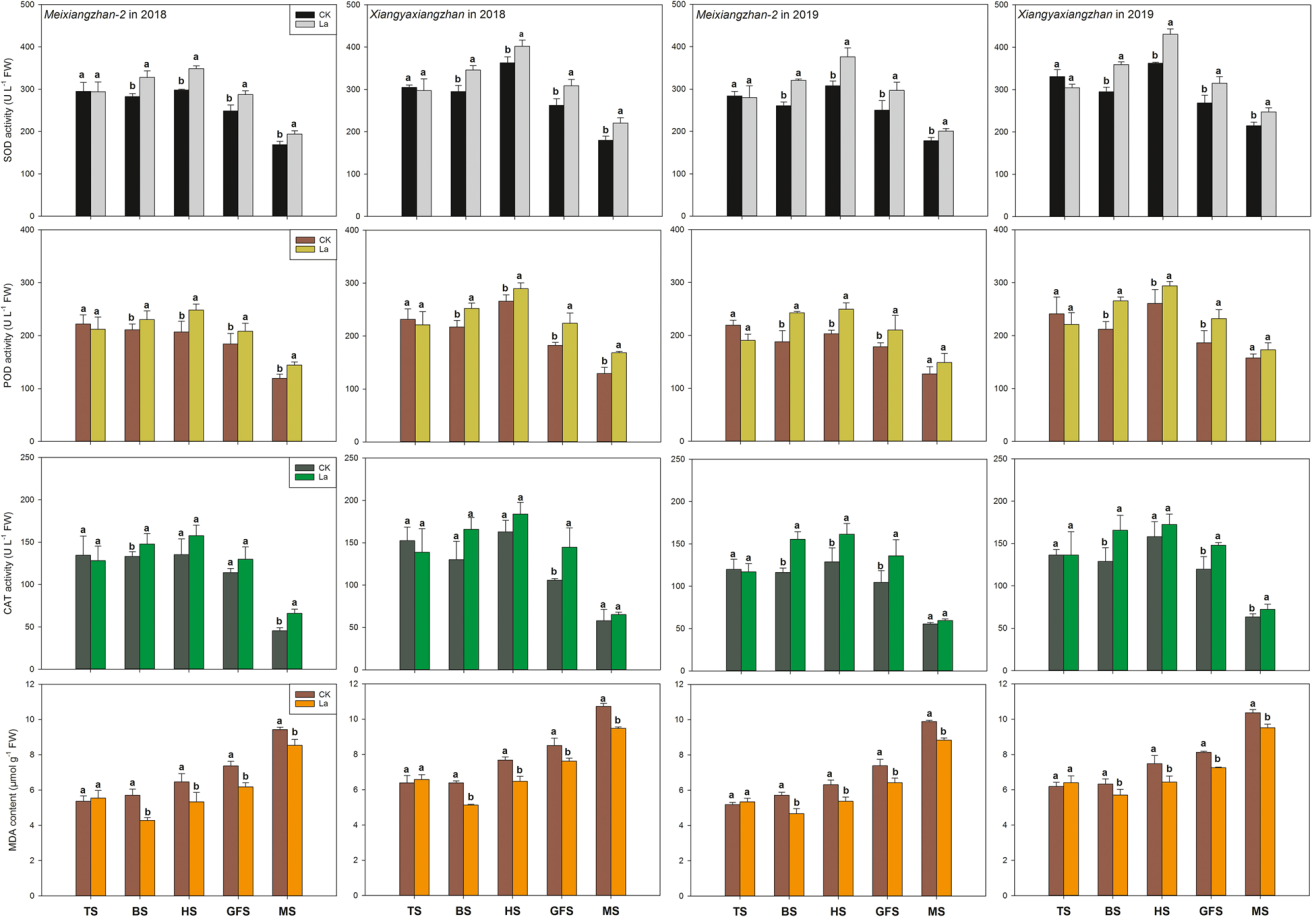
Effects of La application on the activities of SOD, POD, and CAT and the contents of MDA in aromatic rice in the third experiment. TS: tillering stage; BS: booting stage: HS: heading stage: GFS: grain-filling stage; MS: maturity stage. Values (means ± SEs) of each treatment were obtained from three independent replications (n = 3). Different letters indicate significant differences among the treatments (*P* < 0.05, least significant difference test)

As shown in Fig. 7, La application affected the MDA content and induced regulation in antioxidative enzyme (SOD, POD and CAT) activity. There was no significant difference between CK and the La treatment in the activities of SOD, POD, and CAT or the content of MDA at the tillering stage. However, compared with CK, the La treatment increased the activities of SOD, POD and CAT by 10.86–23.08%, 8.89–24.44% and 7.14–36.37%, respectively, from the booting stage to the maturity stage. The MDA contents were 8.26–24.83% lower than in the La treatment than CK from the booting stage to the maturity stage.

### Effects of La application on 2-AP content and 2-AP biosynthesis in aromatic rice

**Fig. 8:**
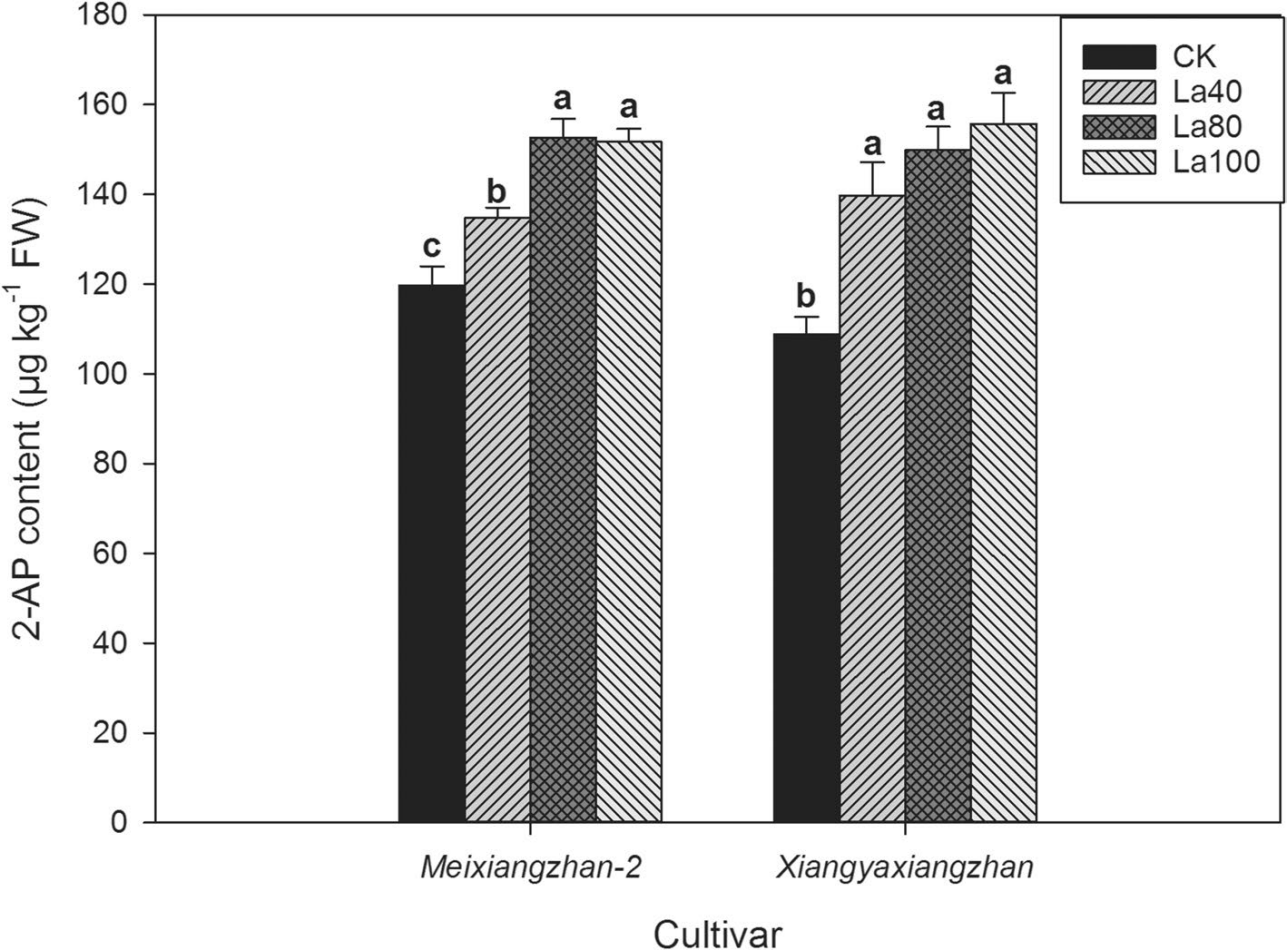
Effects of different La treatments on the 2-AP content of aromatic rice in the second experiment. Values (means ± SEs) of each treatment were obtained from three independent replications (n = 3). Different letters indicate significant differences among the treatments (*P* < 0.05, least significant difference test)

**Fig. 9:**
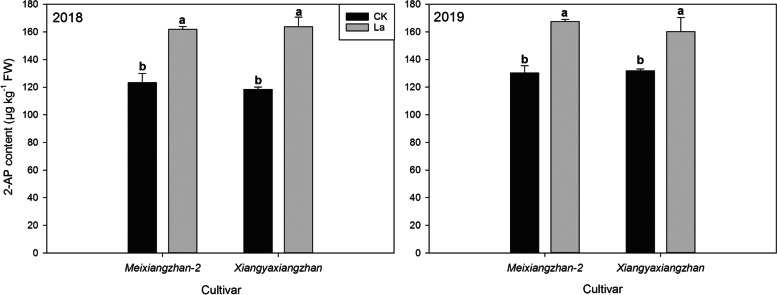
Effects of La application on the 2-AP content of aromatic rice grains in the third experiment. Values (means ± SEs) of each treatment were obtained from three independent replications (n = 3). Different letters indicate significant differences among the treatments (*P* < 0.05, least significant difference test)

**Fig. 10:**
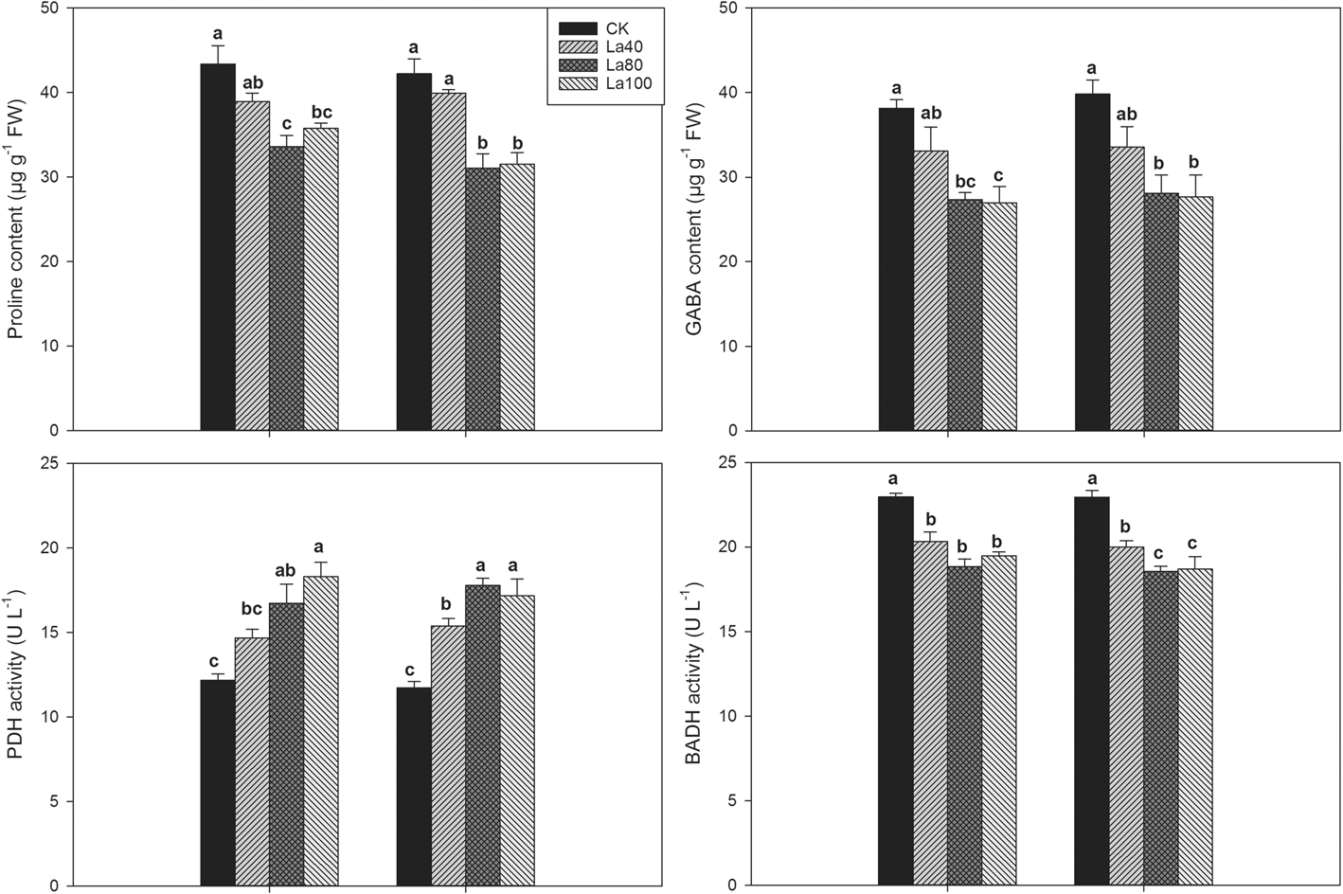
Proline content, GABA content, PDH activity and BADH activity of aromatic rice under La application in the second experiment. Values (means ± SEs) of each treatment were obtained from three independent replications (n = 3). Different letters indicate significant differences among the treatments (*P* < 0.05, least significant difference test)

In the second experiment, aromatic rice plants grown in La containing soil showed higher grain 2-AP contents (Fig. 8, Fig. 9, Fig. 10 and Table 4). For *Meixiangzhan-2*, the La40, La80, and La100 treatments substantially increased the grain 2-AP contents by 12.61, 27.53, 26.72% compared with those in CK; For *Xiangyaxiangzhan*, 28.36, 37.62, and 43.03% higher 2-AP contents were recorded in the La40, La80, and La100 treatments than CK. As shown in Fig. 10, La application regulated 2-AP biosynthesis in terms of the proline, and GABA contents and, PDH and BADH activity. In comparison with CK, La80 and La100 treatments significantly reduced the grain contents of proline and GABA. Higher PDH activity was recorded in the La80 and La100 treatments than in CK. Lower BADH activity was recorded in the La80 and La100 treatments than in CK.

**Table 4:** Proline content, GABA content, PDH activity and BADH activity of aromatic rice under La application in the field in the third experiment

Year	Cultivar	Treatment	Proline (μg g^−1^ FW)	GABA (μg g^−1^ FW)	PDH activity (U L^−1^ FW)	BADH activity (U L^−1^ FW)
2018	Meixiangzhan-2					
		CK	43.14 ± 1.37a	32.19 ± 1.52a	13.45 ± 0.67b	22.48 ± 0.35a
		La	34.12 ± 0.43b	25.63 ± 1.10b	17.58 ± 0.72a	18.54 ± 0.43b
	Xiangyaxiangzhan					
		CK	40.59 ± 0.62a	35.73 ± 1.15a	13.46 ± 1.15b	21.01 ± 0.45a
		La	31.19 ± 0.53b	28.08 ± 1.71b	18.27 ± 1.07a	17.13 ± 0.84b
2019	Meixiangzhan-2					
		CK	41.98 ± 0.84a	34.51 ± 0.23a	12.04 ± 0.91b	21.51 ± 0.39a
		La	33.21 ± 1.20b	24.12 ± 1.57b	18.89 ± 1.27a	17.39 ± 0.59b
	Xiangyaxiangzhan					
		CK	43.31 ± 2.67a	39.16 ± 1.28a	13.23 ± 1.09b	20.99 ± 0.28a
		La	32.77 ± 1.71b	25.89 ± 2.54b	16.40 ± 0.32a	18.59 ± 0.25b

Values (means ± SEs) of each treatment were obtained from three independent replications (n = 3). Different letters indicate significant differences among the treatments (*P* < 0.05, least significant difference test)

In the field production (the third Experiment), La application to the soil markedly increased the 2-AP content in aromatic rice grains (Fig. 9). In 2018, compared with CK, the La treatment significantly increased the grain 2-AP content by 31.36 and 32.85% in *Meixiangzhan-2* and *Xiangyaxiangzhan*, respectively. In 2019, in comparison with CK, the La treatment significantly increased the grain 2-AP content by 28.55 and 23.08% in *Meixiangzhan-2* and *Xiangyaxiangzhan*, respectively. As shown in Table 4, compared with CK, the La treatment remarkably reduced the contents of proline and GABA, and the activity of BADH. Higher PDH activity was recorded in the La treatment than in CK.

## Discussion

Crop growth and development are substantially affected by La, this has been previously reported in several plant species, such as wheat (*Triticum aestivum* L.), maize (*Zea mays* L.) and soybean (*Glycine max* (Linn.) Merr.)[16–18]. The current study demonstrated the modulatory effects of La application to soil on 2-AP biosynthesis in aromatic rice. In the first experiment, we observed that aromatic rice seedlings grown in La containing soil (80 and 100 LaCl_3_ mg kg^−1^) not only had higher fresh weight, dry weight, plant height and stem length, but also had higher 2-AP contents than those grown in soil without La. In both the second and third experiments, the La treatments also substantially increased the grain yield of the aromatic rice cultivars. The increment in grain yield due to La application was attributed to improvement in grain number per panicle and seed-setting rate. Our results were consistent with the study of [19] who demonstrated that exogenous La significantly increased rice yield. As the method of dry matter accumulation in plants, photosynthesis was significantly influenced by La application in terms of the chlorophyll content and net photosynthetic rate in aromatic rice. The results of the present study showed that La treatments remarkably increased SPAD values and enhanced the net photosynthetic rate of the aromatic rice cultivars, *Meixiangzhan-2* and *Xiangyaxiangzhan* at the booting stage, heading stage, grain-filling stage and maturity stage. The total aboveground biomass at the booting stage, heading stage, grain-filling stage and maturity stage (total yield) also increased due to La application. Therefore, we deduced that La enhanced yield formation in aromatic rice by regulating photosynthesis and dry matter accumulation.

In our study, higher activities of antioxidant enzymes (SOD, POD, CAT) and lower MDA content were observed in aromatic rice under the La treatments. Previous studies indicated the important roles of SOD, POD and CAT play in maintaining cellular structures and functions, and quenching reactive oxygen species in plant tissues [20, 21]. These enhancements in antioxidant activity indicated that La might improve ability of aromatic rice to resist different abiotic stresses. In the field, variations in climate especially the sudden occurrence of short-term extreme weather, have substantial effects on rice yield [22]. Moreover, the microclimate in paddy fields which is complicated and difficult to measure, also influences yield formation of rice [23]. A stronger antioxidant system in rice ensures stable rice production stabilization. Furthermore, chlorophyll biosynthesis and gas exchange for photosynthesis are affected by the environment [20, 24] while higher antioxidant enzyme activity can better maintain the stability of cells and ensure the progress of various physiological activities [25, 26]. Therefore, we deduced that the application of La might protect chlorophyll biosynthesis in aromatic rice by increasing the activities of antioxidant enzymes and that this would enhance photosynthesis and dry matter accumulation and finally increase grain yield.

Regarding 2-AP, a key component of the aroma of aromatic rice, the La treatments increased the grain 2-AP content and decreased the grain proline content in the second experiment. A similar result was also observed in the La treatment in the third experiment. Furthermore, the results of both the second and third experiments showed that the activity of PDH was significantly enhanced in the La treatments; this result is similar to the research of Mo et al. [10], who indicated that the additional La in culture medium increased the activity of PDH and the 2-AP content in aromatic rice panicles in vitro. The function of PDH is to catalyze the oxidation reaction of proline to convert △1-pyrroline-5-carboxylic acid, which is an important precursor for 2-AP biosynthesis [27, 28]. Therefore, the increase in grain 2-AP content in aromatic rice could be attributed to exogenous La enhancing PDH activity and thus leading to the promotion of the conversion from proline to 2-AP.

**Fig. 11:**
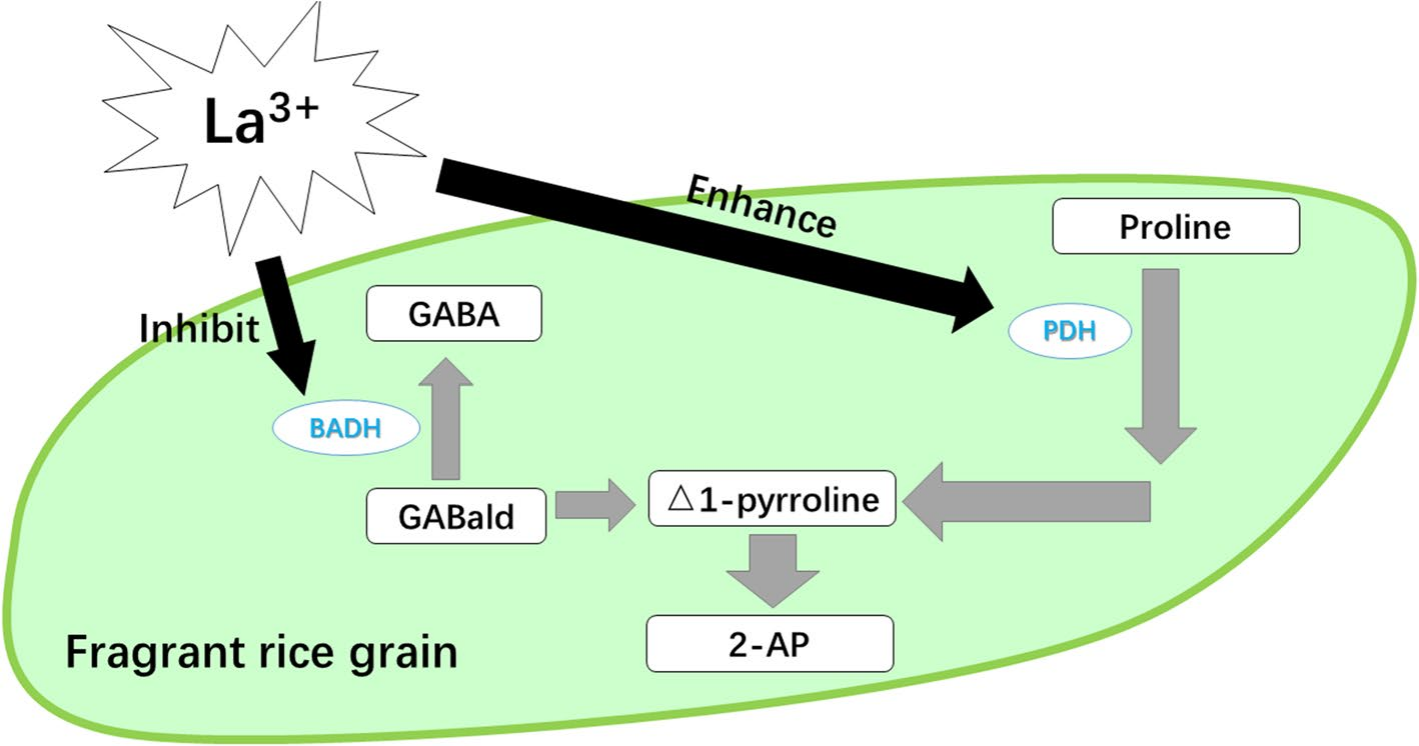
The potential roles of exogenous La in regulation of 2-AP biosynthesis in aromatic rice plants

In addition, the present study revealed that lower grain proline and GABA contents were recorded in the La treatments than in CK in both the second and third experiments. In 2-AP biosynthesis in aromatic rice, a competitive relationship exists between GABA and 2-AP and the conversion from GABald to △1-pyrroline (a limited substrate in 2-AP formation [6]) or GABA depends on the reaction catalyzed by the enzyme BADH [9, 29, 30]. The results of the second and third experiments showed that the activity of BADH declined under the La treatments. Therefore, we deduced that exogenous La also increases the conversion from GABald to Δ1-pyrroline by inhibiting GABA formation, thus increasing the 2-AP content in aromatic rice. The possible mechanism for the regulation of grain 2-AP biosynthesis in aromatic rice by exogenous La is shown in Fig. 11.

## Conclusion

The application of La to soil increased the chlorophyll content and antioxidant enzyme activities of aromatic rice. Increases in the net photosynthetic rate, dry matter accumulation and yield formation were observed in the La treatments. La application also improved the conversion from proline to 2-AP by upregulating the activity of the enzyme PDH and thus increased the grain 2-AP content. In addition, La application inhibited BADH activity and thus reduced the conversion of GABald to GABA during 2-AP biosynthesis in aromatic rice.

## Methods

### Experimental details and plant materials

To study the effect of La on 2-AP biosynthesis in aromatic rice, three experiments (two pot experiments and a two-year field experiment) were conducted with two aromatic rice cultivars, *Meixiangzhan-2* (*Lemont* × *Fengaozhan*) and *Xiangyaxiangzhan* (*Xiangsimiao126* × *Xiangyaruanzhan*). These cultivars are widely planted in South China, and were provided by the College of Agriculture, South China Agricultural University, Guangzhou, China. Detailed information on the varieties could be found at https://ricedata.cn/. The three experiments were performed as described below:

The first experiment was conducted at the College of Agriculture, South China Agricultural University, Guangzhou, China between October and December 2017. After soaking and germination, the seeds of aromatic rice were sown in plastic pots (14.5 cm upper diameter, 10.5 cm lower diameter and 7.5 cm height) filled with paddy soil. There were five La treatments i.e., La1: 20 mg kg^−1^ LaCl_3_; La2: 40 mg kg^−1^ LaCl_3_; La3: 80 mg kg^−1^ LaCl_3_; La4: 100 mg kg^−1^ LaCl_3_; and La5: 120 mg kg^−1^ LaCl_3_. Pots without applied La were used as controls (CK). Twenty days after sowing, the seedlings were collected and the fresh weight, dry weight, plant height, stem diameter and 2-AP content were determined.

The second experiment was conducted in the greenhouse of the Experimental Research Farm, College of Agriculture, South China Agricultural University, Guangzhou, China (23°09’N, 113°22’ E and 11 m above the sea level) between March and July 2018. The germinated seeds of aromatic rice were sown in soils contained in plastic nursery trays. When the seedlings were twenty days old, they were transplanted into pots with five hills per pot (32 cm upper diameter, 21 cm lower diameter and 23 cm height) and four seedlings per hill. According to the results of the first experiment, three La treatments were implemented, i.e., La40: 40 mg kg^−1^ LaCl_3_; La80: 80 mg kg^−1^ LaCl_3_; and La100: 100 mg kg^−1^ LaCl_3_. Pots without La application were used as the controls (CK). At the grain-filling and maturity stages, fresh panicles were collected and stored at -80 °C for biochemical and molecular analysis. The grain yield and yield-related traits of aromatic rice were also estimated at the maturity stage.

The third experiment was conducted at the Experimental Research Farm, South China Agricultural University, Ningxi County (23°16’N, 113°22’E and 11 m above sea level), Guangdong Province, China between July and November in 2018 and again in 2019. After germination in the nursery, fifteen-day-old seedlings were mechanically transplanted into paddy fields at a planting distance of 30 cm × 16 cm. According to the results from both the first experiment and the second experiment, the La treatment was set as 12 kg ha^−1^ LaCl_3_, and a treatment without La application was used as a control (CK). The treatments were arranged in a randomized complete block design (RCBD) in triplicate with a total plot size of 10 m × 3 m. At the tillering stage (20 days after transplanting), booting stage (40 days after transplanting), heading stage (60 days after transplanting), grain-filling stage (75 days after transplanting) and maturity stage (90 days after transplanting, also 1 day before harvesting), fresh flag leaves from each plot were collected and stored at -80℃. At the grain-filling stage and maturity stage, fresh panicles were also collected and stored at -80℃ for biochemical analysis. The grain yield and yield related traits of aromatic rice were also estimated at the maturity stage.

### Determination of net photosynthetic rate, chlorophyll content and dry matter accumulation

At the tillering stage (20 days after transplanting), booting stage (40 days after transplanting), heading stage (60 days after transplanting), grain-filling stage (75 days after transplanting) and maturity stage (90 days after transplanting, also 1 day before harvesting) in the second and third experiments, the net photosynthetic rate was determined with the portable photosynthesis system (LI-6400, LI-COR, USA) according to the method described by Luo et al. (2019). At the same time, a ‘SPAD-502’ SPAD meter (Konica Minolta, Japan) was used to perform a precise, rapid and nondestructive estimation of the leaf chlorophyll contents. In the second experiment, the total aboveground tissue of aromatic rice from three random pots in each treatment was collected at the maturity stage and oven-dried at 80 °C for the determination of the total yield. In the third experiment, plants from 9 randomly selected hills were collected at the tillering stage, booting stage, heading stage, grain-filling stage and maturity stage and oven-dried at 80 °C to a constant weight for the determination of the total aboveground biomass.

### Determination of activities of superoxide (SOD), peroxidase (POD), catalase (CAT) and contents of malondialdehyde (MDA)

The MDA content and activities of antioxidant enzymes in the leaves of aromatic rice were determined according to the methods described by Kong et al. [20]. The MDA content was detected after a reaction with thiobarbituric acid while the absorbance was read at 532 nm, 600 nm and 450 nm. The activities of SOD, POD and CAT were determined and expressed as U L^−1^ FW.

### Determination of 2-AP content

The 2-AP contents in the grain samples collected at maturity and the seedling samples were determined using the simultaneous distillation–extraction method (SDE) and analyzed by a GCMS-QP 2010 Plus (Shimadzu Corporation, Japan) according to the methods of [31].

### Determination of the contents of proline and GABA, and activities of PDH and BADH

The contents of proline and GABA in the grain samples collected at the grain-filling stage were determined according to the methods described by Mo et al. [4]. The proline content was determined after reacting with sulfosalicylic acid and the absorbance was measured at 520 nm. The GABA in the samples was extracted using 60% ethanol, and the absorbance was read at 645 nm. The GABA content was expressed as μg g^−1^ FW. The activities of PDH and BADH in grains at the grain-filling stage were estimated according to the methods of Luo et al. [2]. For BADH activity, the absorbance was read at 450 nm and expressed as U L^−1^. For PDH activity, the absorbance was read at 440 nm and expressed as U L^−1^.

### Statistical analyses

All data were analyzed statistically using Statistix 8 (Analytical software, Tallahassee, Florida, USA) with one-way analysis of variance. The differences among means were separated by using the least significant difference (LSD) test at the 5% significance level.

## Data Availability

All data generated or analysed during this study are included in this published article.

## Abbreviations

2-AP: 2-Acetyl-△1-pyrroline
BADH: Betaine aldehyde dehydrogenase
CAT: Catalase
GABA: γ- Aminobutyric acid
La: Lanthanum
MDA: Malondialdehyde
PDH: Proline dehydrogenase
POD: Peroxidase
SOD: Superoxide dismutase
